# Comparison of a blood based mitochondrial biomarker with plasma ATN performance

**DOI:** 10.1002/alz70856_106233

**Published:** 2026-01-08

**Authors:** Brittany M Hauger, Keith P Smith, Taylor A. Strope, Ellen Oehmke, Paul J Kueck, Riley E Kemna, Casey S. John, Leonidas Bantis, Jill K Morris, Russell H Swerdlow, Heather M Wilkins

**Affiliations:** ^1^ University of Kansas Alzheimer's Disease Research Center, Kansas City, KS, USA; ^2^ 3901 rainbow blvd, Kansas City, KS, USA; ^3^ University of Kansas Medical Center, Kansas City, KS, USA; ^4^ University of Kansas Alzheimer's Disease Research Center, Fairway, KS, USA; ^5^ University of Kansas Alzheimer's Disease Center, Fairway, KS, USA; ^6^ Department of Neurology, University of Kansas Medical Center, Kansas City, KS, USA; ^7^ University of Kansas Medical Center, Alzheimer's Disease Research Center, Fairway, KS, USA; ^8^ Department of Biochemistry and Molecular Biology, University of Kansas Medical Center, Kansas City, KS, USA; ^9^ Department of Cell Biology and Physiology, University of Kansas Medical Center, Kansas City, KS, USA; ^10^ Department of Neurology, University of Kansas School of Medicine, Kansas City, KS, USA

## Abstract

**Background:**

Alzheimer's disease (AD) pathology begins decades before clinical onset of dementia. Amyloid beta (Aβ) generally accumulates first in cognitively normal (ND) individuals, with tau and cognitive abnormalities following. AD pathologies have been found to correlate and interact with metabolic and mitochondrial outcomes in studies spanning numerous experimental paradigms. Targeting metabolic and mitochondrial function in clinical trials is an emerging theme for AD and highlights the importance and need for biomarkers which measure mitochondrial function.

**Method:**

20 ND, 20 mild‐cognitive impairment, and 19 Alzheimer's Disease (AD) subjects were recruited from the KU Alzheimer's Disease Research Center Clinical Cohort. Blood was collected in ACD tubes. Lymphocytes were isolated using Accuspin tubes, histopaque 1077, and centrifugation. Lymphocytes were stained with MitoTracker/Annexin V, MitoSox/Hoechst, and TMRE/Hoechst. Fluorescent cells were quantified using flow cytometry and values normalized to Hoechst. Fluorescent measures were completed within 30 hours of blood draw. The mitochondrial health index (MHI) algorithm was calculated. Plasma NfL, pTau181, Aβ, and GFAP were determined using SIMOA. *APOE* genotypes were determined using a Taqman allelic discrimination assay.

**Result:**

The MHI biomarker was significantly reduced in AD subjects. In MCI subjects, the MHI predicted a later reduction in mini mental state exam (MMSE) scores. *APOE ε4* carriers had reduced MHI regardless of diagnosis. Plasma GFAP, NfL, and pTau181 were increased in AD subjects. ROC analysis for individual biomarkers for ND/AD: Aβ 0.6658, GFAP 0.7194, NfL 0.7194, pTau181 0.7796, MHI 0.8028. ROC analysis for individual biomarkers for ND/MCI: Aβ 0.6700, GFAP 0.5737, NfL 0.5368, pTau181 0.7105, MHI 0.6025. ROC analysis for individual biomarkers for MCI/AD: Aβ 0.5632, GFAP 0.5965, NfL 0.6462, pTau181 0.5078, MHI 0.8139. Combining the MHI with ATN in an algorithm improved ROC, however including Aβ in the algorithm worsened ROC.

**Conclusion:**

The MHI biomarker is a blood‐based biomarker with reasonable discrimination for AD diagnosis. We are currently examining if the MHI correlates with brain glucose metabolism outcomes. Mitochondrial biomarkers are urgently needed to assess therapeutic validity in upcoming clinical trials.

